# Advancing Our Understanding of Depression—From Molecular Mechanisms to Novel Therapeutic Frontiers

**DOI:** 10.3390/ijms27188069

**Published:** 2026-09-10

**Authors:** Terezia Kiskova-Simkova

**Affiliations:** Department of Pathology, Faculty of Medicine, Pavol Jozef Safarik University in Kosice, 040 01 Kosice, Slovakia; terezia.simkova@upjs.sk

Major Depressive Disorder (MDD), often termed the “silent epidemic” of the 21st century, affects millions of individuals worldwide [1]. While clinical symptoms like persistent sadness and anhedonia are well-recognized, the true depth of the condition lies in a complex web of molecular alterations within the brain [2]. This Special Issue is designed to highlight research that bridges the gap between basic molecular neuroscience and the development of more effective, personalized therapeutic interventions.

The landscape of modern psychiatry is shifting from a symptom-based diagnostic approach toward mechanism-based stratification and precision therapeutics [3]. This evolution is driven by the integration of multi-omics, neuroimaging, and advanced computational algorithms that aim to identify biologically grounded disease subtypes [4]. A prime example of this multi-system etiology is the microbiota-gut-brain (MGB) axis, which influences neuropsychiatric health by modulating tryptophan-kynurenine metabolism, blood-brain barrier integrity, and central neuroinflammatory pathways [5]. Microbial dysbiosis can trigger chronic neuroinflammation via microglial activation and the NLRP3 inflammasome, contributing to functional brain connectivity deficits [5]. Exploring therapeutic approaches that target this axis, such as precision psychobiotics and bioactive plant molecules, helps bridge the gap between basic neurobiology and clinical intervention, directly aligning with plant-derived therapies evaluated in this collection. Furthermore, understanding these systemic interactions is crucial when addressing sex-specific differences in depression, where females exhibit distinct neurobiological, immune, and transcriptional signatures [5].

Recent research into adolescent major depressive disorder (AMDD) utilizes the multimodal integration of morphometric similarity networks (MSN) and spatial transcriptomics to delineate distinct neurobiological subtypes [6]. For instance, one identified subtype (AMDD1) is characterized by frontoparietal network disruptions and deficits in synaptic pruning, while another (AMDD2) is linked to GABAergic inhibition deficits and MAPK-driven neuroinflammation [6]. These subtypes suggest that identical clinical symptoms, such as irritability or anhedonia, may arise from fundamentally different developmental and molecular trajectories.

In our Special Issue, the authors focused on refining our understanding of depression through animal models. Sarkisova and Kudrin investigated sex differences in depression comorbid with absence epilepsy using the WAG/Rij rat model (publication 1; Contribution 1). Their findings reveal that while both sexes exhibit depression-like behaviors, females experience more pronounced anhedonia and anxiety, linked to more severe dopamine insufficiency and altered serotonin metabolism. Complementing this, Szabó et al. (Contribution 2) explored the prolactin-releasing peptide (PrRP) system in male rats, identifying it as a potential mediator of stress coping mechanisms. Their study found that passive coping strategies are associated with brain-region-specific changes in PrRP mRNA expression, particularly in the medullary A1 region and the habenula (Figure 1).

A neuroactive metabolite of progesterone, allopregnanolone (ALLO), is synthesized in the brain and peripheral tissues and is an important modulator of GABAα receptors. By enhancing GABAergic inhibitory neurotransmission, ALLO regulates neuronal excitability, stress responses, emotional processing, and potentially, neuroplasticity. Its concentrations fluctuate during the menstrual cycle and increase markedly during pregnancy, followed by a rapid decline after delivery. Alterations in ALLO synthesis, metabolism, or receptor sensitivity have therefore been proposed as potential mechanisms contributing to vulnerability to mood disorders, particularly during hormonally sensitive periods [7].

Evidence from animal models indicates that manipulation of ALLO synthesis or signaling can influence stress-related and depressive-like behaviors, while restoration of neurosteroid signaling may produce antidepressant-like effects [8,9]. Human studies measuring ALLO have also reported associations between altered ALLO levels and depressive symptoms [10,11]. However, findings remain inconsistent, potentially due to differences in sex, reproductive status, menstrual cycle phase, age, medication exposure, sampling compartment, and analytical methods. Thus, ALLO cannot yet be considered a universally reliable biomarker of depression.

Modern molecular psychiatry is increasingly focusing on the regulation of gene expression [12]. It is known that the heritability estimate of MDD is 30–40%, suggesting that genetics alone does not account for most of the risk of major depression. Recent studies have demonstrated that the biological effects of environmental factors in MDD and other stress-related disorders are mediated, at least in part, by a range of epigenetic modifications. These alterations contribute to dysregulated neuroendocrine responses, impaired neuroplasticity, disrupted neurotransmission, and neuroglial dysfunction, all of which play key roles in the pathophysiology of MDD. Moreover, specific epigenetic signatures have been associated with both the diagnosis of MDD and treatment response. Therefore, investigating epigenetic modifications offers valuable insights into the heterogeneous etiology and complex clinical manifestations of MDD and may facilitate the identification of novel biomarkers and therapeutic targets [13].

Modi et al. (Contribution 3) introduced a novel method for targeted DNA methylation using single-stranded methylated DNA probes (Figure 2). By targeting the FKBP5 and MAOA genes, they successfully increased DNA methylation and attenuated the upregulation of these genes in response to stress hormones. This approach offers a simple and potentially safer alternative to CRISPR-based technologies for treating stress-related psychiatric disorders. Furthermore, Chen et al. (Contribution 4) employed single-nucleus transcriptomic analysis to explore male-specific synaptic plasticity in MDD patients. They identified specific excitatory neuron clusters and hub genes like CASKIN1, which are regulated by miR-21-5p. The study suggests that elevated miR-21-5p levels may drive the decreased neural connectivity and altered plasticity characteristic of MDD, providing new insights into the disorder’s cellular dynamics.

Neuroinflammation has emerged as a central biological link in depression. Research has shown that patients with MDD exhibit increased immune activity, including enhanced lymphocyte proliferation and elevated production of pro-inflammatory cytokines such as interleukins IL-1β, IL-6, and interferon gamma (IFNγ), accompanied by increased acute-phase proteins and neuroendocrine activation. These findings were further supported by numerous studies reporting elevated circulating inflammatory markers, including cytokines, soluble cytokine receptors, C-reactive protein (CRP), neopterin, and prostaglandins, particularly in individuals with treatment-resistant depression. Additional investigations revealed alterations in specific inflammatory pathways, including increased TNFα, IL-8, IL-18, and activation of the NLRP3 inflammasome, as well as disruptions in the circadian regulation of immune responses. Overall, current evidence indicates that immune dysregulation and persistent low-grade inflammation contribute significantly to the pathophysiology of MDD, although the strength of these associations may depend on factors such as sex, age, adiposity, and other biological or clinical variables [14].

A systematic review and meta-analysis by Gędek et al. (Contribution 5) evaluated cytokine levels in first episode and drug-naïve patients (Figure 3). They confirmed significant elevations in IL-6, IL-2, and TNF-α, highlighting that immune-inflammatory dysregulation is present at the earliest stages of the disorder, independent of prior medication effects. This inflammatory dysregulation also has profound implications for emergency psychiatry. Lewandowska et al. (Contribution 6) summarized findings on inflammatory biomarkers as predictors of acute suicidal behavior. They noted that markers such as C-reactive protein (CRP), IL-6, and various hematological ratios (e.g., NLR, PLR) could serve as objective adjuncts to clinical evaluation, though their utility is currently limited by a lack of standardized cut-off values.

Natural compounds exhibit multifaceted therapeutic neuroprotective effects by targeting multiple interconnected pathophysiological processes [15]. In this Special Issue, Urbanska et al. (Contribution 7) provide a comprehensive review of the therapeutic potential of selected plants and their bioactive molecules in managing depression and anxiety. These plant-derived compounds, such as polyphenols, flavonoids, and alkaloids, are becoming increasingly recognized as viable treatment options [16] due to their ability to modulate postnatal neurogenesis—a process with significant implications for cognitive and neurological health [17]. By stimulating neural stem cell proliferation, enhancing synaptic plasticity, and reducing neuroinflammatory signaling, these natural agents present promising, low-toxicity adjuvant strategies that complement traditional monoaminergic antidepressant therapies [18,19,20].

To address the persistent challenge of treatment-resistant depression, non-invasive and biological brain stimulation interventions are receiving renewed focus. Selakovic et al. (Contribution 8) systematically review the neurobiological mechanisms of transcranial magnetic stimulation (TMS) in MDD. The authors emphasize how TMS-induced electric fields stimulate the brain-derived neurotrophic factor (BDNF) system and its high-affinity receptor TrkB, triggering intracellular signaling cascades that promote structural remodeling, dendritic spine growth, and synaptic plasticity in prefrontal brain regions.

Complementing this, Fetahovic et al. (Contribution 9) examined electroconvulsive therapy (ECT) from a molecular perspective. Despite historical stigma, ECT remains one of the most effective interventions for severe treatment-refractory depression. The authors outline how ECT drives its rapid antidepressant effects by dramatically boosting central BDNF expression, modulating the hyperactive hypothalamic-pituitary-adrenal (HPA) axis, reducing chronic circulating neuroinflammatory markers, and restoring mitochondrial bioenergetics. Together, these reviews (Contribution 8, Contribution 9) demonstrate that physical brain stimulation and pharmacological approaches converge on shared neuroplastic mechanisms to restore functional neural connectivity.

The contributions to this Special Issue emphasize that depression is not a single entity but a spectrum of molecularly distinct states. From the sex-specific dopaminergic tone in epilepsy models to the potential of targeted epigenetic editing, as well as the neuroprotective effects of plant-based molecules, these findings point toward a future of precision psychiatry. By identifying objective biomarkers and novel molecular targets, we move closer to individualized treatment strategies.

In the near future, the integration of these biological insights with emerging data-driven stratification methods, including advanced computational and artificial intelligence approaches to biomarker discovery, will be paramount. Leveraging machine learning models to analyze multi-omics and neuroimaging datasets represents the next frontier in precision psychiatry, promising to translate complex molecular profiles into objective, clinical-grade diagnostic subtypes.

## Figures and Tables

**Figure 1:**
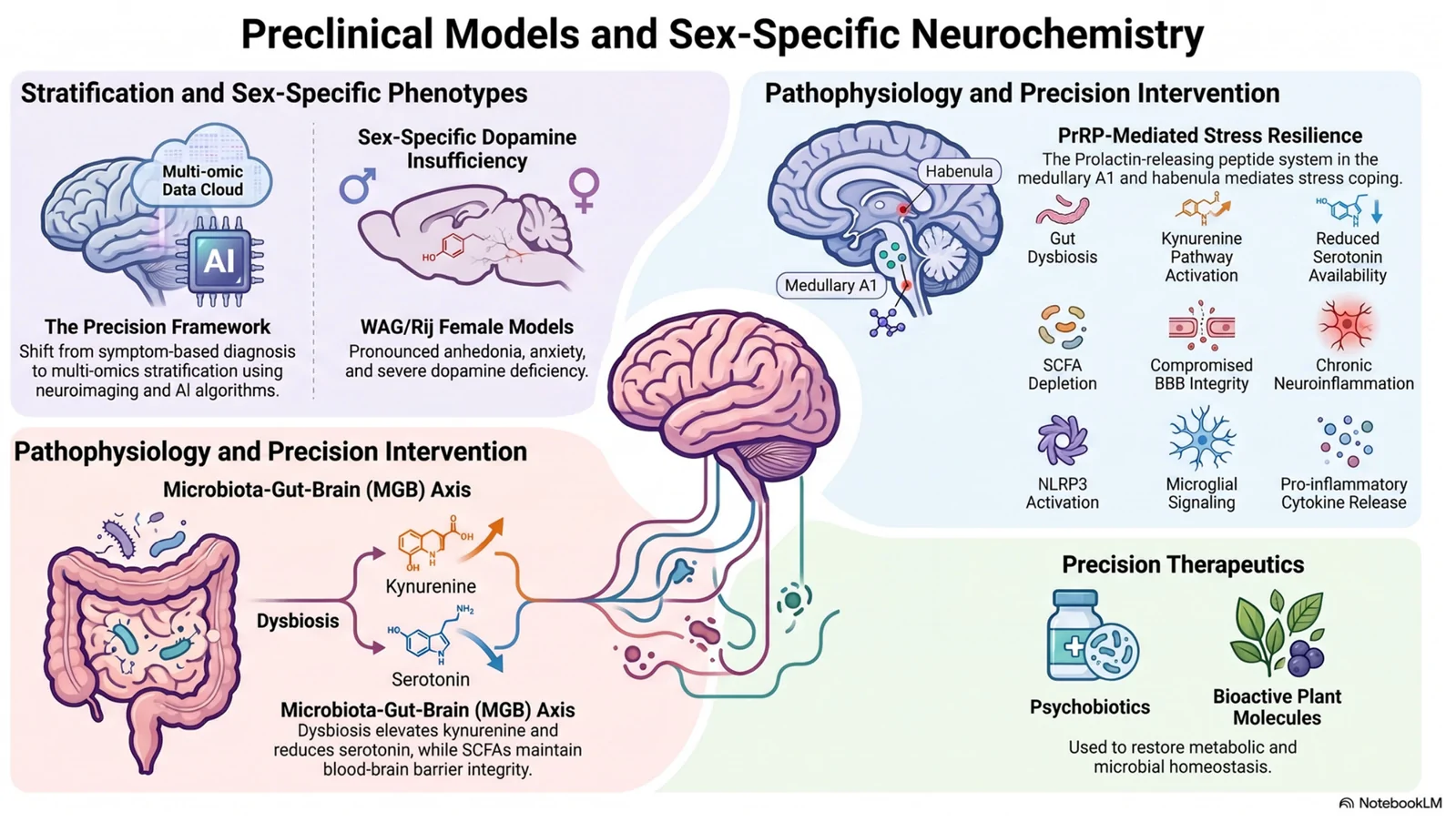
Key biological mechanisms underlying modern precision psychiatry. The figure illustrates the integration of multi-omics approaches and advanced computational tools to support mechanism-based stratification of psychiatric disorders. It highlights the microbiota–gut–brain axis, sex-specific neurobiological differences, stress-coping mechanisms involving the prolactin-releasing peptide (PrRP) system, and emerging precision therapeutic strategies, including psychobiotics and bioactive plant-derived compounds.

**Figure 2:**
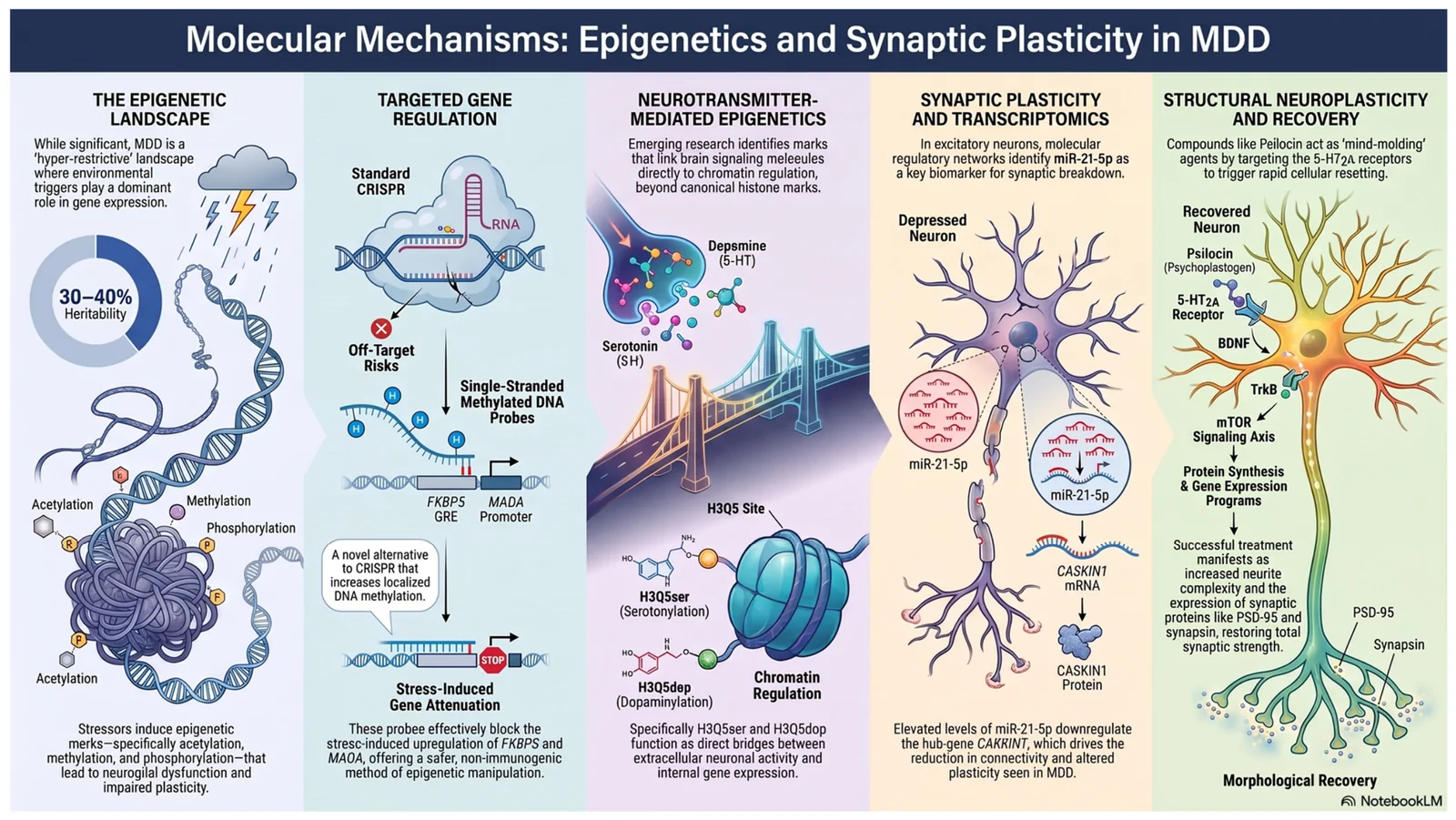
Molecular mechanisms involved in depression, highlighting the interplay between epigenetic regulation and synaptic plasticity. The figure summarizes the impact of environmental stressors on epigenetic modifications, targeted gene regulation, neurotransmitter-mediated histone modifications, transcriptomic alterations affecting neuronal connectivity, and neuroplasticity-promoting mechanisms underlying therapeutic recovery.

**Figure 3:**
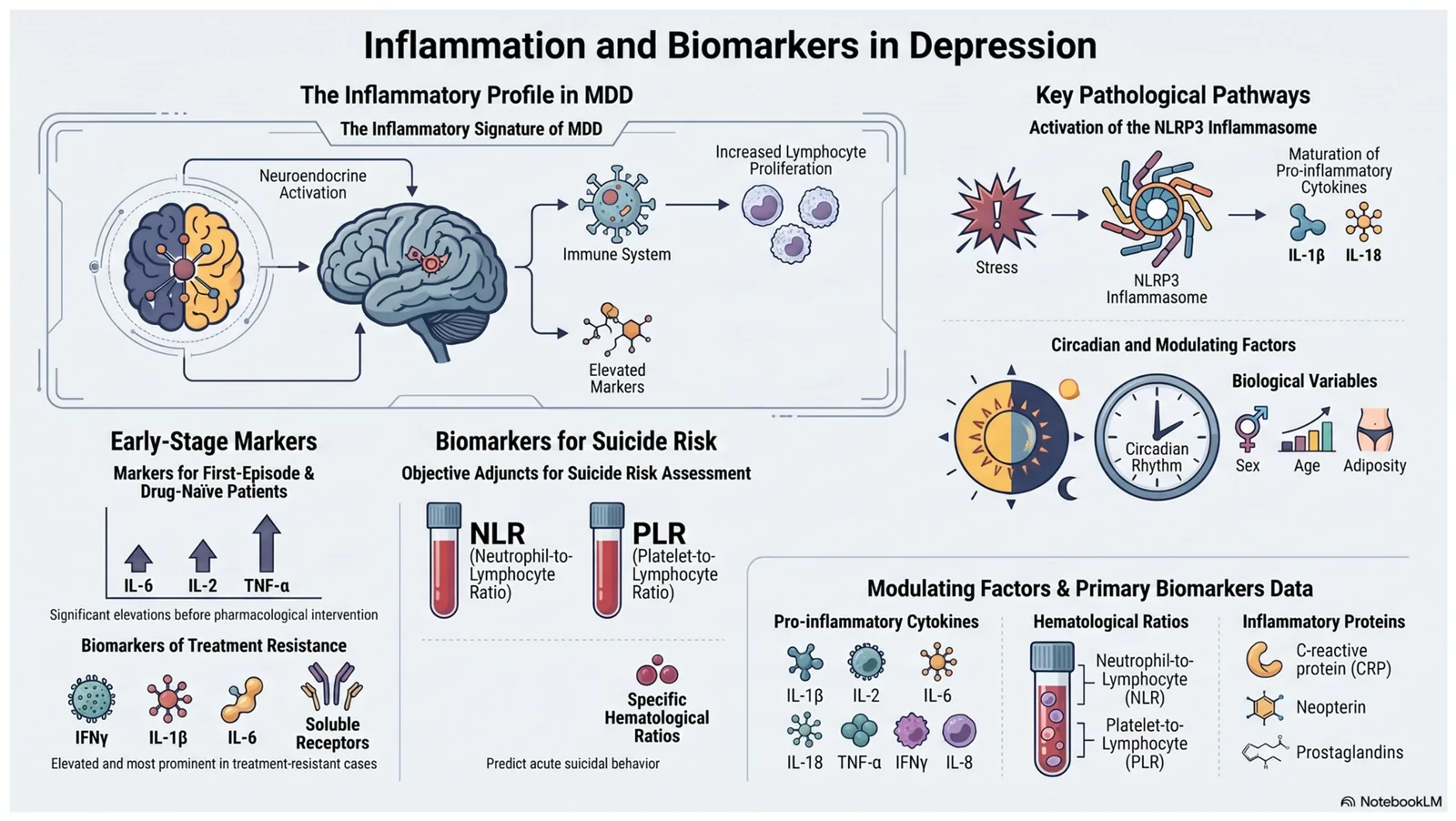
Immune dysregulation and inflammatory biomarkers associated with major depressive disorder. The figure summarizes key inflammatory pathways, early immune alterations, biomarkers associated with suicide risk, and the influence of biological factors such as sex, age, and adiposity on the inflammatory profile of depression.
